# Regulation of Neuroinflammation: What Role for the Tumor Necrosis Factor-Like Weak Inducer of Apoptosis/Fn14 Pathway?

**DOI:** 10.3389/fimmu.2017.01534

**Published:** 2017-11-16

**Authors:** Audrey Boulamery, Sophie Desplat-Jégo

**Affiliations:** ^1^Aix-Marseille University, CNRS, NICN, Marseille, France; ^2^AP-HM, Hôpital Sainte-Marguerite, Centre Antipoison et de Toxicovigilance, Marseille, France; ^3^Service d’Immunologie, Hôpital de la Conception, Marseille, France

**Keywords:** tumor necrosis factor-like weak inducer of apoptosis, Fn14, central nervous system, neuroinflammation, multiple sclerosis, neuropsychiatric systemic lupus erythematosus, stroke, glioma

## Abstract

Observed in many central nervous system diseases, neuroinflammation (NI) proceeds from peripheral immune cell infiltration into the parenchyma, from cytokine secretion and from oxidative stress. Astrocytes and microglia also get activated and proliferate. NI manifestations and consequences depend on its context and on the acute or chronic aspect of the disease. The tumor necrosis factor-like weak inducer of apoptosis (TWEAK)/Fn14 pathway has been involved in chronic human inflammatory pathologies such as neurodegenerative, autoimmune, or malignant diseases. New data now describe its regulatory effects in tissues or fluids from patients with neurological diseases. In this mini-review, we aim to highlight the role of TWEAK/Fn14 in modulating NI in multiple sclerosis, neuropsychiatric systemic lupus erythematosus, stroke, or glioma. TWEAK/Fn14 can modulate NI by activating canonical and non-canonical nuclear factor-κB pathways but also by stimulating mitogen-activated protein kinase signaling. These downstream activations are associated with (i) inflammatory cytokine, chemokine and adhesion molecule expression or release, involved in NI propagation, (ii) matrix-metalloproteinase 9 secretion, implicated in blood–brain barrier disruption and tissue remodeling, (iii) astrogliosis and microgliosis, and (iv) migration of tumor cells in glioma. In addition, we report several animal and human studies pointing to TWEAK as an attractive therapeutic target.

## Introduction

Initially described as an accumulation of leukocytes in multiple sclerosis (MS) brain, neuroinflammation (NI) now also applies to other central nervous system (CNS) diseases (1). Among others factors, it results from peripheral immune cell infiltration through the brain barriers, from cytokine secretion and from oxidative stress (2). NI is based upon and is regulated by bidirectional communication pathways involving especially cytokines that connect the CNS and immune system (3). However, using the generic term “NI” for such a multifaceted process could be inaccurate (1). NI features depend on disease-specific conditions and differ in CNS infection, ischemia, and malignant or autoimmune diseases. Whether NI is acute or chronic is a major point to consider. In fact, transient NI is usually beneficial and must be preserved, while chronic NI is preferentially associated with diseases resulting in neurodegeneration (4, 5).

Neuroinflammation highly involves cellular components of the neurovascular unit (6, 7), which consists in microvascular endothelial cells surrounded by basal lamina, astrocytic end-feet, pericytes, and neurons. It underlies the concept of a blood–brain barrier (BBB) actively separating the parenchyma from the circulation. Resident CNS cells such as astrocytes and microglia are also involved in regulating NI and respond to CNS insults by a proliferation and an abnormal activation, respectively called astrogliosis and microgliosis. This response is not always a harmful process, it can also be a beneficial and crucial process involved in CNS repair (8–10). During NI, immune cells, microglia, and astrocytes release soluble proteins, called cytokines, which mediate cell–cell communication (11). Among them, the tumor necrosis factor (TNF)-like weak inducer of apoptosis (TWEAK) plays a dual role in the physiological versus inflammatory pathological responses of tissues, including the CNS (12–15).

Tumor necrosis factor-like weak inducer of apoptosis is a member of TNF superfamily initially shown to induce apoptosis of malignant cells (16). It is synthesized as a transmembrane protein form (mTWEAK) and proteolytically processed as a soluble cytokine (sTWEAK). Monocytes/macrophages are the main source of sTWEAK in inflammatory tissues. Until now, mTWEAK has only been described on freshly isolated monocytes (17, 18). Unlike TNF-α, TWEAK curtails the innate immune response and attenuates the transition to adaptive Th1 immunity (19). Moreover, TWEAK inhibits TNF receptor-1 signaling that promotes inflammation (20). TWEAK signaling mainly requires binding to fibroblast growth factor inducible 14 (Fn14), a member of the TNF receptor superfamily. Although Fn14 is poorly expressed in healthy endothelial cells, neurons, astrocytes, microglia, and progenitor cells, it is highly inducible in these cells. TWEAK interaction with its Fn14 receptor induces multiple molecular events and biological responses depending on cell type and microenvironment. These downstream signaling pathways have been compiled from the literature in 2012 by Bhattacharjee et al. (21), who cataloged 46 proteins and 28 induced genes. Thus, Fn14 engagement primarily activates nuclear factor-κB (NF-κB) and mitogen-activated protein kinase (MAPK) *via* interaction with intracellular TNF-receptor-associated-factors (21, 22). mTWEAK seems to activate more efficiently the canonical NF-κB pathway while both membrane and soluble TWEAK can induce the non-canonical NF-κB pathway (23). Secondary TWEAK signaling pathways have also been described, such as the phosphatidylinositol 3-kinase/Akt pathway (14).

In the CNS, TWEAK targets endothelial cells, astrocytes, and neurons. Interestingly, in murine and human astrocytes, the TWEAK/Fn14 pathway can stimulate reactivity, i.e., when cells proliferate, are activated and produce inflammation factors (24, 25). This associated mitogenic potency is mediated by the TWEAK-induced MAPK signaling pathway (26). Besides, in an *in vitro* model of human BBB, TWEAK induced microvascular cerebral endothelial cells to display an inflammatory profile: they increased (i) their secretion of proinflammatory cytokines, (ii) their production and activation of matrix-metalloproteinase 9 (MMP-9) involved in BBB disruption, and (iii) their expression of intercellular adhesion molecule-1 implicated in leukocyte adhesion to endothelium (27).

Here, we aimed to review TWEAK role in NI modulation in neurodegenerative, autoimmune, ischemic, and malignant CNS inflammatory diseases. Considering available data about TWEAK involvement in their pathogenesis, we will successively focus on MS, neuropsychiatric systemic lupus erythematosus (NPSLE), cerebral ischemia, glioma, amyotrophic lateral sclerosis (ALS), Parkinson’s disease, and schizophrenia (Table 1).

**Table 1:** Mechanisms underlying the role of tumor necrosis factor-like weak inducer of apoptosis (TWEAK) in neuroinflammation regulation.

Mechanisms	Model	References
Stimulation of immune cell recruitment in central nervous system	Murine model of MS; murine model of NPSLE; murine model of cerebral ischemia	(31, 40, 47)
Stimulation of astrogliosis and microgliosis	Murine model of ALS; astrocytes culture; murine model of NPSLE	(15, 26, 40)
Attenuation of transition from innate to adaptative immunity with Th1 helper decrease	Murine model of TWEAK-KO	(19)
Cellular death	Neuronal *in vitro* model of ischemia; murine model of ALS; postmortem MS brains; murine model of NPSLE	(13, 15, 29, 40)
Downstream activation of canonical and non-canonical NF-κB pathways	Murine model of TWEAK-KO; *in vitro* model of BBB; murine model of NF-κB p50-KO; human astrocytoma cell cultures	(19, 22, 23, 45, 51, 55)
Downstream activation of MAPK pathway	Astrocyte cultures; *in vitro* model of BBB	(26, 27)
Downstream activation of Rac1 pathway	Human astrocytoma cell cultures	(51, 53)
Increase in VCAM-1 and ICAM-1 production	*In vitro* model of BBB; murine model of NPSLE	(27, 40)
Increase in MMP-9 production and activity	*In vitro* model of BBB; murine model of NF-κB p50-KO; murine model of cerebral ischemia; glioma cell cultures	(27, 45, 46, 55)
Decrease in ZO-1 expression	*In vitro* model of BBB	(27)
Increase in synthesis of pro-inflammatory mediators Il-6, Il-8, RANTES, and CCL-2	Murine model of ALS; *in vitro* model of BBB; murine model of NPSLE; murine model of cerebral ischemia	(15, 27, 37, 39, 40, 46, 47)
Cerebral Ig G deposition, increase in albumin quotient and decrease in aquaporin-4 expression	Murine model of NPSLE	(37, 39, 40)
Activation of complement and iNOS	Murine model of NPSLE	(37, 39, 40)

*ALS, amyotrophic lateral sclerosis; BBB, blood–brain barrier; CCL-2, chemokine ligand 2; ICAM-1, intercellular adhesion molecule-1; Il, interleukin; Ig, immunoglobulin; Inos, inducible form of nitric-oxide synthase; KO, knock-out; MAPK, mitogen-activated protein kinase; MMP-9, matrix-metalloproteinase 9; MS, multiple sclerosis; NF-κB, nuclear factor-κB; NPSLE, neuropsychiatric systemic lupus erythematosus; Rac1, Ras-related C3 botulinum toxin substrate 1; RANTES, regulated on activation, normal T Cell expressed and secreted; VCAM-1, vascular cell adhesion molecule-1; ZO-1, zonula occludens-1*.

## The TWEAK/Fn14 Pathway Contributes to Tissue Injury in MS

Multiple sclerosis is a multifactorial disease involving auto-immunity against myelin components, NI, BBB disruption, tissue remodeling, and demyelination/remyelination (28).

### TWEAK and Fn14 Are Upregulated in MS

Serafini et al. showed that both TWEAK and Fn14 were upregulated in postmortem MS brain sections (29). Furthermore, this increase was related to the degree of inflammation and demyelination. Perivascular and intrameningeal macrophages, microglia, and astrocytes were the main sources of TWEAK, with a different contribution according to lesion location and degree of inflammation. Fn14 was expressed by neurons and astrocytes in the cortex of highly infiltrated MS brains (29). The absence of TWEAK/Fn14 expression in healthy brain reinforces the idea that TWEAK/Fn 14 pathway could play a role in MS pathogeny. In blood, Desplat-Jégo et al. demonstrated that mTWEAK was expressed at the cell surface of monocytes derived from MS patients but not from non-MS patients (18). In MS, such mTWEAK-expressing monocytes could represent immune cells that infiltrate CNS by interacting with Fn14 molecules at the membrane of BBB endothelial cells. In the same study, sTWEAK serum and cerebrospinal fluid levels were similar in MS and non-MS patients (18), suggesting that sTWEAK is not a reliable diagnosis MS biomarker.

Myelin oligodendrocyte glycoprotein-induced experimental autoimmune encephalomyelitis (EAE) is the best-characterized animal model of MS. In chronic EAE induced in C57Bl/6 mice, TWEAK transcript levels increased in the spinal cord (24). Moreover, transgenic mice overexpressing sTWEAK developed a more severe EAE than wild-type mice (24). Besides, in cuprizone-treated mice, a model of demyelination/remyelination, TWEAK and Fn14 transcription were upregulated during both demyelination and remyelination phases (30).

### Blocking the TWEAK/Fn14 Pathway Is Effective for Treating an MS Model

Treated with cuprizone TWEAK-knock-out mice displayed a significant delay in microglia accumulation and in demyelination phases (30). Reinforcing the role of TWEAK/Fn14 in MS, our team showed that EAE severity and CNS leukocyte infiltration were reduced in mice treated with blocking monoclonal anti-TWEAK antibody after the priming phase (31). Moreover, an Fn14-TNF-related apoptosis-inducing ligand (TRAIL) fusion protein was designed to simultaneously block endogenous TWEAK and mediate TRAIL inhibitory signals on activated T cells (32). Injected in EAE-mice, this protein reduced EAE severity (32, 33). Additionally, vaccinating mice or rats with TWEAK extracellular domain or with Fn14 induced the production of specific inhibitory antibodies. Such treatment was associated with reduced inflammatory spinal cord infiltration and with clinical amelioration during EAE (34). However, in humans, a raising question is the relevance of targeting TWEAK in MS when anti-TNF-α has failed to improve disease status (35). One could argue that the TWEAK/Fn14 pathway could also play a role in neuroprotection. In this way, Iocca et al. observed a modest but significant delay in remyelination of TWEAK-knock-out mice treated with cuprizone. However, this marginal delay did not result in prolonged defect in remyelination (30). Later, Echeverry et al. described a TNF-α-dependent neuroprotective effect of TWEAK in another NI model (13), but data in MS are lacking. Further studies are then needed to definitively establish that TWEAK is a relevant therapeutic target in MS.

## The TWEAK/Fn14 Pathway Contributes to NPSLE

Neuropsychiatric systemic lupus erythematosus is an autoimmune disease underlied by hyperactivation of B and T lymphocytes, leading to overproduction of autoantibodies, tissue deposition of immune complexes, and increase in proinflammatory cytokines. This inflammatory state potentially targets all the organs, with marked joint, renal, hematologic and skin damages. In a significant number of patients, neuropsychiatric manifestations can also occur and thus define NPSLE. A pathogenic role of TWEAK was shown in lupus nephritis, a renal manifestation of the disease (36). Besides, evidences of TWEAK involvement in NPSLE are growing.

### TWEAK/Fn14 Interaction Compromises the BBB in NPSLE Mouse Model

Putterman et al. observed an increased expression of TWEAK and Fn14 in the cerebral cortex of the MRL/lpr mouse strain with NPSLE (37, 38). Furthermore, knocking out Fn14 in this strain (i) significantly improved cognitive function and (ii) decreased depression and anhedonia in comparison with MRL/lpr wild-type mice. These clinical parameters were associated with decreased levels of proinflammatory cytokines and preserved BBB integrity (37). Further, CNS anatomopathological studies in these mice showed (i) a reduction of fibronectin deposition and inducible form of nitric-oxyde synthase (iNOS) production, (ii) a diminution of immune infiltrates, and (iii) a decrease of IgG deposition and complement activation (39). Finally, reduced cortex neuronal degeneration, apoptosis, and gliosis were associated with improved cognitive functions in such mice (40).

### TWEAK Mediates Symptoms Observed in NPSLE

These results were consolidated by intracerebroventricular injection of Fc-TWEAK in non-autoimmune mice. Just like MRL/lpr strain, these mice developed NPSLE symptoms, associated with high levels of TWEAK pathway downstream components, complement and iNOS activation, IgG brain deposition, neurodegeneration, and apoptosis (39). Trysberg et al. suggested that NPSLE could be associated with high levels of MMP-9 and sTWEAK in cerebrospinal fluid (41, 42). They also found a significant correlation between intrathecal MMP-9 and levels of tau (a neuronal degeneration marker) and glial fibrillary acidic protein (an astrocytic degeneration marker) (41). These results suggest that TWEAK/Fn14 interaction might be involved in brain damages during NPSLE since it promotes synthesis and activation of MMP-9 in CNS (27). Nevertheless, a recent study concluded that TWEAK levels in serum and cerebro-spinal fluid did not seem relevant biomarkers for NPSLE (43).

Finally, these findings highly support the involvement of TWEAK/Fn14 interaction in NPSLE pathogenesis and symptom occurrence.

## TWEAK Displays a Dual Role in Cerebral Ischemia

Cerebral ischemia or stroke is the second cause of mortality in the world. The onset of cerebral ischemia is followed by inflammatory events that affect neurological patient’s outcome. Several studies implicate TWEAK in stroke.

### TWEAK/Fn14 Interaction Increases the Volume of the Ischemic Zone in Stroke

Experimental focal cerebral ischemia was associated with an increase in TWEAK and Fn14 mRNA levels (44) and with BBB disruption (45, 46). In this context, TWEAK-induced NF-κB activation resulted in both astrocytic chemokine (C-C motif) ligand-2 (CCL-2) expression, leading to polynuclear neutrophil recruitment into the damaged zone, and in upregulation of MMP-9 activity (45–47). The effective inhibition of this neuroinflammatory process by Fn14-Fc decoy injections (46, 48) was associated with a reduced ischemic zone and hastened motor function recovery (46). Stroke patients displayed significantly elevated TWEAK serum levels and, in postmortem stroke human brains, elevated Fn14 mRNA levels were associated with upregulated Fn14 immunostaining in the ischemic zone (49).

### The TWEAK/Fn14 Signaling Contributes to the Protective Effect of Hypoxic Preconditioning

Hypoxic-preconditioning of mice or cultured neurons promotes neuron survival and reduces ischemic lesion volume. In this context, TWEAK and Fn14 levels increase. Moreover, the protective effect of hypoxic preconditioning was abrogated in TWEAK or Fn14 KO mice and restored in cultured neurons after addition of TWEAK (13). This suggests that the TWEAK/Fn14 pathway contributes to the protective effect of hypoxic preconditioning. This is likely mediated by neuronal TNF-α and the activation of the MAPK pathway, resulting in the inactivation of the Bcl-2-associated death promoter protein (13).

These results suggest that a relevant therapeutic strategy would be administration of TWEAK in order to protect the brain in patients at high risk of stroke.

## TWEAK Activates Canonical and Non-Canonical NF-κB Pathways in Glioblastoma

Glioblastoma, the most common primary brain tumor, is associated with a high mortality related to its high local invasiveness, angiogenesis, and immunosuppressive potency. The initiation and development of tumors are associated with repetitive tissue damage and are tightly linked to chronic inflammatory processes. Tumor cell migration is essential in glioblastoma malignancy progression and depends on its inflammatory microenvironment.

### TWEAK/Fn14 Promotes Glioblastoma Cell Migration and Invasion

Tumor necrosis factor-like weak inducer of apoptosis upregulates Fn14 in migrating glioblastoma cells. This overexpression correlates with tumor aggressiveness and poor outcome (50, 51). In this way, Fn14 expression may help classifying glioma histological subtype (52). Moreover, canonical NF-κB pathway, stimulated by TWEAK, promotes Rac1 and Cdc42 expression, key mediators in the regulation of glioblastoma cell migration and invasion (51, 53, 54). Interestingly, Cherry et al. showed that MMP-9 inhibition abolished TWEAK-induced glioma invasion (55).

### Targeting Fn14 Is Promising for Treating Glioblastoma

Therapeutic strategies targeting Fn14 in malignant cells have been evaluated. In these preclinical studies, manipulating Fn14 expression levels or suppressing TWEAK-Fn14-NFκB-dependent signaling decreased glioblastoma cell invasion capacity (56, 57). However, further experiments are needed to evaluate whether these strategies can significantly impact patients’ survival.

## The TWEAK/Fn14 Pathway is Involved in NI-Associated Neurodegeneration in ALS

Amyotrophic lateral sclerosis is a fatal neurodegenerative disease affecting motor neurons in the brain and spinal cord, and associated with NI hallmark (astrogliosis and microgliosis) and skeletal muscle atrophy. Mutations within the superoxide dismutase 1 (SOD1) are sometimes found in familial or sporadic cases. Mutant SOD1 expression was associated with inflammatory astrocytes and microglia and with skeletal muscle atrophy (58).

### TWEAK/CD163 Interaction Induces Motor Neuron Death in a Mouse Model of ALS

Bowerman et al. used a model of transgenic mice overexpressing human mutant SOD1 and recapitulating the main traits of ALS to study TWEAK/Fn14 involvement in ALS (15). In this model, mTWEAK was upregulated in the spinal cord and it induced astrocyte proliferation and IL-6 release. TWEAK and Fn14 were also found in skeletal muscle with different patterns of expression linked to disease development. In this study, the authors demonstrated a TWEAK induced-motor neuron death (15). Surprisingly, it was mediated by TWEAK interaction with CD163, a putative alternative TWEAK receptor. This neuronal death involved caspase-3 activation and was totally independent of Fn14. This work is the first implicating CD163 in mediating TWEAK effects in an inflammatory neurological disease. CD163 is a member of the scavenger receptors and is exclusively expressed by monocytes and macrophages. Increasing *in vivo* and *in vitro* evidences suggest that the interaction between TWEAK and CD163 may affect the development of atherosclerosis and related diseases (59).

### TWEAK Promotes Astrogliosis and Microgliosis in ALS

Tumor necrosis factor-like weak inducer of apoptosis deletion reduced astrogliosis and microgliosis in the spinal cord, which substantiated the implication of the TWEAK pathway in ALS. However, muscle pathology was only partially reduced and TWEAK deletion did not prevent motor deterioration nor increase life span. In the same way, anti-TWEAK antibodies injected in ALS mice decreased microgliosis without improving motor functions. Bowerman et al. thus proposed a model combining the action of peripheral and central TWEAK, which could both contribute to the sustained activation of microglia in ALS (15).

This work suggests that blocking TWEAK in ALS is interesting but not sufficient and requires being included in a combinatory therapeutic approach.

## TWEAK Contribution to Parkinson’s Disease and Psychiatric Diseases Needs Confirmation

### Anti-TWEAK Antibodies Improve a Murine Model of Parkinson’s Disease

Parkinson’s disease is a neurodegenerative pathology characterized by movement impairment due to dopaminergic loss in the nigrostriatal pathway. It may also have a neuroinflammatory component, where NF-κB may play a dual role, both promoting and protecting against neurodegeneration (60). The role of TWEAK itself has been studied in the 1-methyl-4-phenyl-1,2,3,6-tetrahydropyridine (MPTP) murine model and in Parkinson’s disease human brain samples (61). There was no difference in substantia nigra TWEAK concentration between MPTP mice, Parkinson’s disease patients, and controls. While TWEAK or Fn14-KO failed to prevent MPTP toxicity, anti-TWEAK antibodies treatment attenuated the MPTP-induced death of dopaminergic neurons (61). These apparently conflicting results could be explained in part by existing compensatory systems, including the TNF-α pathway, since TNF is expressed in microglia of Parkinson’s disease patients.

### TWEAK Plasma Levels in Schizophrenia and Bipolar Disorders Are Not Conclusive

Neuroinflammation is highly suspected to be involved in psychiatric pathologies like schizophrenia or bipolar disorder. Recently, TWEAK plasma levels of patients suffering from schizophrenia were compared with controls. Although no difference was shown globally, TWEAK levels in male patients were significantly lower than in male controls, suggesting TWEAK involvement in subgroups of schizophrenia patients (62). In bipolar disorder, conflicting results were observed, perhaps due to the heterogeneity in patients’ groups: Cingi et al. measured lower TWEAK plasma levels compared to healthy controls (63), while Barbosa et al. described increased concentrations, regardless of the mood (64). Nevertheless, as the interest for studying TWEAK/Fn14 pathway in psychiatric diseases is very recent, further studies will certainly be available soon.

This mini-review reveals that the TWEAK/Fn14 pathway modulates NI in neurodegenerative, immune, ischemic, and malignant CNS inflammatory diseases. In fact, as supported by both animal and human *in vitro* and *in vivo* studies, TWEAK/Fn14 can modulate NI by activating canonical and non-canonical NF-κB pathways but also MAPK signaling. Then, expression or release of inflammatory cytokines, chemokines, and adhesion molecules are upregulated and astrogliosis and microgliosis occur. Additionally, MMP-9 secretion is stimulated and reinforces BBB disruption and tissue remodeling. Note that monoclonal anti-TWEAK antibodies have been injected in patients in phase I clinical studies. In healthy volunteers or patients with solid tumors or rheumatoid arthritis, they (i) displayed a classical therapeutic antibody pharmacokinetic pattern, (ii) were associated with a favorable safety profile, and (iii) induced a decrease in circulating TWEAK serum level (65–68). Additionally, in these studies, TWEAK/Fn14 pathway inhibition yielded encouraging results, such as reduced clinical and biological inflammatory markers, including sTWEAK. All these data support the concept that the TWEAK/Fn14 axis represents a promising therapeutic target for modulating inflammation, including NI.

## Author Contributions

AB and SD-J both collected the publications and wrote the manuscript.

## Conflict of Interest Statement

The authors declare that the research was conducted in the absence of any commercial or financial relationship that could be construed as a potential conflict of interest.
